# Light Emission from Plasmonic Nanostructures Enhanced with Fluorescent Nanodiamonds

**DOI:** 10.1038/s41598-018-22019-z

**Published:** 2018-02-26

**Authors:** Jingyi Zhao, Yuqing Cheng, Hongming Shen, Yuen Yung Hui, Te Wen, Huan-Cheng Chang, Qihuang Gong, Guowei Lu

**Affiliations:** 1 State Key Laboratory for Mesoscopic Physics & Collaborative Innovation Center of Quantum Matter, Department of Physics, Peking University, Beijing, 100871 China; 2 Institute of Atomic and Molecular Sciences, Academia Sinica, Taipei, 104 Taiwan, China; 3 Collaborative Innovation Center of Extreme Optics, Shanxi University, Taiyuan, Shanxi 030006 China

## Abstract

In the surface-enhanced fluorescence (SEF) process, it is well known that the plasmonic nanostructure can enhance the light emission of fluorescent emitters. With the help of atomic force microscopy, a hybrid system consisting of a fluorescent nanodiamond and a gold nanoparticle was assembled step-by-step for *in situ* optical measurements. We demonstrate that fluorescent emitters can also enhance the light emission from gold nanoparticles which is judged through the intrinsic anti-Stokes emission owing to the nanostructures. The light emission intensity, spectral shape, and lifetime of the hybrid system were dependent on the coupling configuration. The interaction between gold nanoparticles and fluorescent emitter was modelled based on the concept of a quantised optical cavity by considering the nanodiamond and the nanoparticle as a two-level energy system and a nanoresonator, respectively. The theoretical calculations reveal that the dielectric antenna effect can enhance the local field felt by the nanoparticle, which contributes more to the light emission enhancement of the nanoparticles rather than the plasmonic coupling effect. The findings reveal that the SEF is a mutually enhancing process. This suggests the hybrid system should be considered as an entity to analyse and optimise surface-enhanced spectroscopy.

## Introduction

Surface-enhanced fluorescence (SEF), enhancing the light emission of fluorescent emitters with metallic nanostructures, is the key to many promising applications^1,2^. By placing the emitters in the vicinity of metallic nanostructures, the luminescence can be greatly enhanced because of the plasmon resonance effect. Metallic nanostructures support localised surface plasmon (LSP) modes, which gives rise to a marked enhancement of local electromagnetic fields and results in a strongly modified local density of optical states^3,4^. Metallic nanostructures known as optical antennas can be involved in the excitation and emission process of the emitters^4–8^. The metallic nanostructures enable the modification of the emission intensity, lifetime, directivity, and spectral shape^5,9–14^. Basic theories of the SEF process have been developed^15–17^. In the conventional theory of SEF, there is a lack of attention on how the fluorescent emitters influence the behaviour of the metallic nanostructures. Most of the previous SEF theories often assume that the light emission of the metal nanostructures is negligible or stable^5,18–20^; this approximation allows us to mostly understand SEF.

The SEF background was often simply subtracted as a stable signal when calculating the SEF enhancement factor. A deviation of the enhancement factor is inevitable if the emission from the metal nanostructures changes owing to the presence of emitters. Furthermore, this approximation often hinders us from fully and accurately understanding the SEF spectral shape^4,21–23^. Intrinsic light emission from metal nanostructures has been demonstrated as a complementary property to absorption and scattering^24–26^. Although the mechanism is still under debate, a solid consensus is that the metal nanostructures can radiate at their LSP resonances after photon or electron excitation^27,28^. Such light emission (here, it is called photoluminescence (PL)) from the nano-metal can be correlated with the broad “background continuum” in the SEF process^29,30^.

The interactions between fluorescent emitters and metallic nanostructures has been extensively investigated experimentally^31,32^. Despite much progress, the changes in the light emission of the plasmonic nanostructures influenced by the fluorescent emitters have often been ignored in previous studies. Access to experimental analysis of light emission of plasmonic nanostructures during the SEF process has also proven elusive for a long time. This problem has not been addressed in many recent studies, probably because of the experimental challenge of isolating the intrinsic emission signal of the metallic nanostructures; the PL of the metallic nanoparticles is usually broad and weak, and which often overlaps with the light emission of fluorescent emitters. As a result, it is nontrivial to observe the changes of the intrinsic emission from the metallic nanoparticles during the SEF process. Here, we found that the difference in the emission between the metal nanostructures and the fluorescent emitters can be distinguished through the anti-Stokes emission, which enabled us to circumvent previous experimental challenges. Specifically, we performed optical measurements at the single particle level to avoid inhomogeneous average-broadened spectra. The light emission from the same gold nanoparticles and nanodiamonds were recorded *in situ* and compared on the same substrate.

In this study, we experimentally demonstrate that the light emission from the gold nanostructures is not a constant signal and is enhanced when couple with fluorescent emitters. Not only can the gold nanostructure enhance the light emission of the fluorescent emitters, as expected, but the emitters can also increase the light emission from the gold nanostructures. With the help of an atomic force microscope (AFM), a single gold nanoparticle was manipulated step-by-step to approach a single fluorescent nanodiamond (FND) on a glass coverslip. The emission spectra excited with a continuous-wave laser were recorded *in situ* and compared before and after coupling. We found that the emission from the gold nanoparticle can be distinguished from the SEF hybrid spectra through its characteristic anti-Stokes emission because the FND does not present any anti-Stokes emission. According to the experimental results, the light emission from the FND and gold nanoparticle both enhanced after coupling. The theoretical calculations reveal that both the plasmonic coupling effect and the dielectric nanoantenna effect contribute to the enhancement of light emission from the nanoparticles. Specifically, the dielectric nanoantenna effect dominates the emission enhancements from the plasmonic nanostructures, as confirmed by correlating the theoretical and experimental results.

## Experimental Results

**Figure 1:**
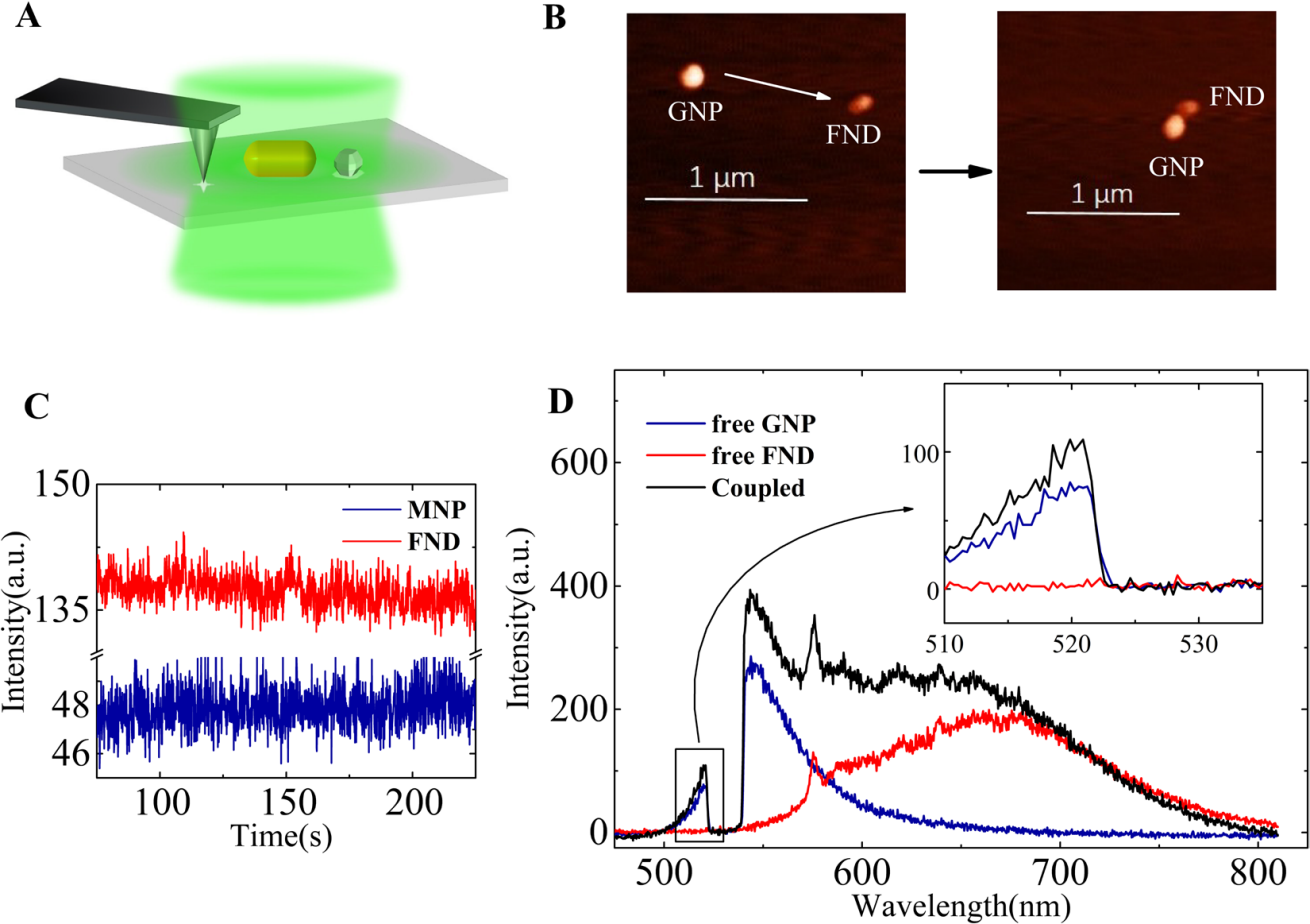
Scheme of nano-manipulation and representative *in situ* optical measurements. (**A**) Scheme of the AFM manipulation method, (**B**) representative AFM images during the assembly process. (**C**) Time trace of the PL intensity of a gold nanoparticle and a nanodiamond. (**D**) The PL spectra of a free GNP (dark blue) and a free FND (red) before coupling and the SEF spectrum (black) after coupling. The inset shows a magnification of the area showing the anti-Stokes component.

We used gold nanoparticles (GNP) and gold nanorods (GNR) to couple with FND for the SEF, as shown in Fig. 1A,B. The representative PL spectrum of a free GNP (Fig. 1D) shows well-defined emission bands rather than the broad continuum band usually associated with a rough thin film, which is a benefit for the analysis of the surface plasmon effect in SEF^33^. The emission spectra of the FNDs and GNPs were both very stable (nonbleaching and nonblinking), as shown in Fig. 1C^34^, which is important for *in situ* comparison before and after coupling. Specifically, as illustrated in Fig. 1D, the emission spectra of the FNDs dominate the range of Stokes components, but do not have any signal in the anti-Stokes range. In contrast, the emission spectra of the GNPs have an obvious anti-Stokes band that decays exponentially as a function of photon energy. The one-photon luminescent anti-Stokes emission of metallic nanostructures has been demonstrated in different metal nanostructures, although the mechanism is still the subject of much debate^27,29,30,33,35^. Interestingly, this anti-Stokes component allows us to differentiate the intrinsic light emission of the GNPs from the SEF hybrid spectra. As shown in the inset of Fig. 1D, the intensity of the anti-Stokes emission from the coupling system increases in comparison with that before coupling. The enhancement factor can be defined as *I*_*c*_/*I*_*uc*_, in which *I*_*c*_and*I*_*uc*_ are the maximum of the PL spectra before and after coupling, respectively. For the anti-Stokes emission, the intensity increased over 40% at a wavelength of 520 nm. There have been numerous reports in the literature about the coupling phenomena of nanodiamonds and plasmonic structures^31,32^, but the anti-Stokes emission has been seldom discussed. Because the anti-Stokes emission is solely owing to the GNP in the present system, this implies that the FND enhances the light emission from the GNPs. The same measurements were performed for the GNR and FND coupling system, as shown in Fig. S1A. The intensity of the spectrum of the coupled GNR and FND system in the Stokes range increased compared to that before coupling, and even compared to the sum of the intensities in the free GNR and FND spectra. The spectral peak of the SEF undergoes a redshift compared to that of the free FND spectrum. For the anti-Stokes component, the intensity of the SEF was also enhanced compared with that of the GNR before coupling. Moreover, the lifetime of the SEF system was also investigated, as shown in Fig. S1B. The lifetime of the free GNR was less than 1 ps, which was faster than the instrument response. The lifetime curves of the free FND can be fitted with two exponential components. Consequently, the lifetime curve of the GNR–FND coupling system contains three exponential components, the coupling between the GNR and FND results in a shorter lifetime of one component owing to the FND. The spectra of the GNRs, FNDs, and their hybrids showed excitation polarisation dependent characteristics as indicated in the supplemental materials. To demonstrate the enhancement of anti-Stokes emission definitely, we measured overall PL spectra of a nanorod before and after coupling with a nanodiamond under different excitation polarisations. Then, we choose two maximum spectra respectively from two series of the spectra (before coupling and after coupling) for comparison, hence, the excitation polarisation effect was excluded indirectly. We also excluded the influences of the excitation power fluctuation and the uncertainty owing to the optical path drifts, which is depicted in Figs S2 and 3. Hence, the SEF phenomena is a mutually enhancing process owing to the interaction between the fluorescent emitters and the gold nanostructures. To calculate the SEF enhancement factor and understand the SEF spectral shape, the change of the light emission from the metal nanostructures must be considered.

**Figure 2:**
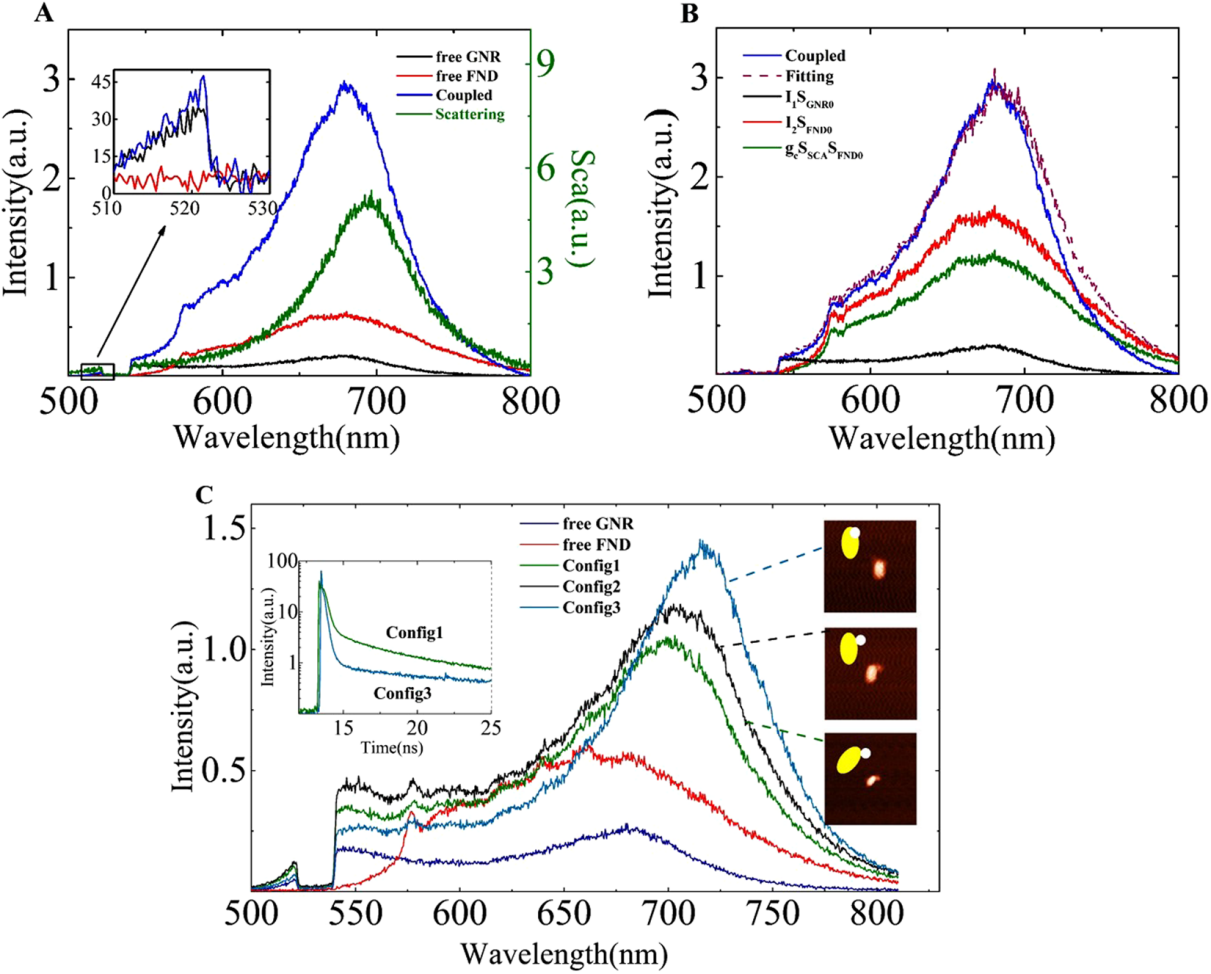
A complete analysis of the three components of the SEF spectrum and coupling-configuration-dependent SEF spectra. (**A**) The PL spectra of a free GNR (black) and a free FND (red) before coupling, the SEF spectrum (blue) and scattering spectrum (green) after the coupling of GNR and FND. (**B**) Fitting spectra for *I*_1_S_*GNR*0_, *I*_2_S_*FND*0_, and ${g}_{c}{\hat{{\rm{S}}}}_{SCA}{\hat{{\rm{S}}}}_{FND0}$. (**C**) Configuration-dependent SEF spectra of the hybrid. The spectra of a free GNR (blue) and a free FND (red) before coupling, and three SEF spectra of three different configurations, as indicated by the AFM and schematic images. The inset shows the lifetime curves of the config. 1 and config. 3 configurations for comparison.

Furthermore, as shown in Figs 1D and 2A, the SEF spectral shape in the Stokes range is totally different from that of free FND or free GNP. It is well-known that the plasmonic nanostructures enable the modification of the emission spectral shape^4^. Ringler *et al*. have developed an empirical formula to correlate the scattering and luminescence spectral shape of GNP dimers and dye molecules systems^11^. Based on their work, we show that the emission from the GNP should be taken into account to fully understand the SEF spectra. We supposed that a SEF spectrum S_*SEF*_ contains three components: two direct emissions S_*GNR*_, S_*FND*_ are from the GNR and FND separately, and one indirect emission (*S*_*gc*_) is owing to the optical antenna coupling effect, i.e., S_*SEF*_ = S_*GNR*_ + S_*FND*_ + *S*_*gc*_. The direct emission S_*GNR*_, based on the following quantum model, is inelastic decay radiation of the plasmon resonance of the GNR. The indirect emission *S*_*gc*_ can be understood as a process of elastic radiation by the antenna after coupling energy from excited states of the fluorescence emitters^11^. We assumed that the coupling rate Γ_*g*_ between the FND and the GNR is less than the internal phonon decay rate of the FND and the frequency-dependent Γ_*g*_ is strongly related to the LSP resonance, i.e., ${{\rm{\Gamma }}}_{g} \sim \,{\hat{{\rm{S}}}}_{SCA}$ (the hybrid scattering spectrum)^6,12,36^. Then, we obtained the three components by fitting the SEF spectrum using ${{\rm{S}}}_{SEF}={I}_{1}{{\rm{S}}}_{GNR0}+{I}_{2}{{\rm{S}}}_{FND0}+{g}_{c}{\hat{{\rm{S}}}}_{SCA}{\hat{{\rm{S}}}}_{FND0}$, in which the terms with a 0 subscript are the spectra before coupling, $\hat{{\rm{S}}}$ is the normalised spectrum, and *I*_1_,*I*_2_,and *g*_*c*_ are the fitting constant parameters. Figure 2A shows the experimental spectra of S_*GNR*0_ and S_*FND*0_ before coupling, and the S_*SEF*_ increasing in both the Stokes and anti-Stokes region after coupling. The fitting results are summarised in Fig. 2B. The direct emission from the GNR and the FND increased by 40% and 140% respectively, and the indirect emission photons by the antenna effect was approximately 43% of the whole SEF spectra. Therefore, the emission from the metallic nanostructures can no longer be omitted or assumed to be constant when calculating the SEF enhancement factor. All three components determine the final SEF spectral shape. However, the integrated intensity of the anti-Stokes emission of the GNR is relatively small; thus, the total modification of the emitted power differs only by a few percent, which indicates that the emission from the metal is necessary to accurately and fully understand the SEF spectral shape. The SEF spectrum of the GNP-FND system shown in Fig. S4 was analysed in the same way as that of the GNR-FND system in Fig. 2B. The SEF spectra was modified by the GNP antenna effect, and the direct emission from the GNP dominated the SEF spectra near the excitation due to the GNP’s LSP resonance band.

In addition, we showed that the SEF spectral shape, intensity, and lifetime vary for different coupling configurations between the GNR and FND^6,37^. The distance between the FND and GNR before coupling was several micrometres, which was enough to isolate them by optics. However, the exact distance between the FND and GNR after coupling could not be determined, with an error of approximately 10 nm. Figure 2C shows the SEF spectra of a GNR and FND hybrid system with a different configuration obtained through AFM nanomanipulations. It is well known that the coupling rate Γ_*g*_ (i.e., being related with the Purcell factor) is dependent on separation and orientation of the emitter with respect to the antenna^36,38^. Then, the indirect emission *S*_*gc*_ intensity is dependent on the coupling strength, i.e., the coupling configuration. The Purcell factor distribution can be similar to the near-field distribution of the GNR^36^. When the FND was closer to the GNR end, the spectral maximum increased and redshifted more, and the lifetime was shorter. Because the coupling strength increased, the indirect emission component increased, which significantly modified the spectral shape. Another factor is that the FND as a dielectric particle would increase the local effective index felt by the GNR, resulting in a redshift of the LSP resonance as well as the SEF spectra^39^. We noted that the fluorescence of nanodiamond is excitation polarisation dependent, the polarisation contributions can be excluded roughly by comparing the spectra of nanodiamond of the same excitation polarisation before and after coupling because the nanodiamonds were always fixed in the experiments. Here, we demonstrate that the SEF process is strongly dependent on the coupling configurations.

## Theoretical Model and Discussions

**Figure 3:**
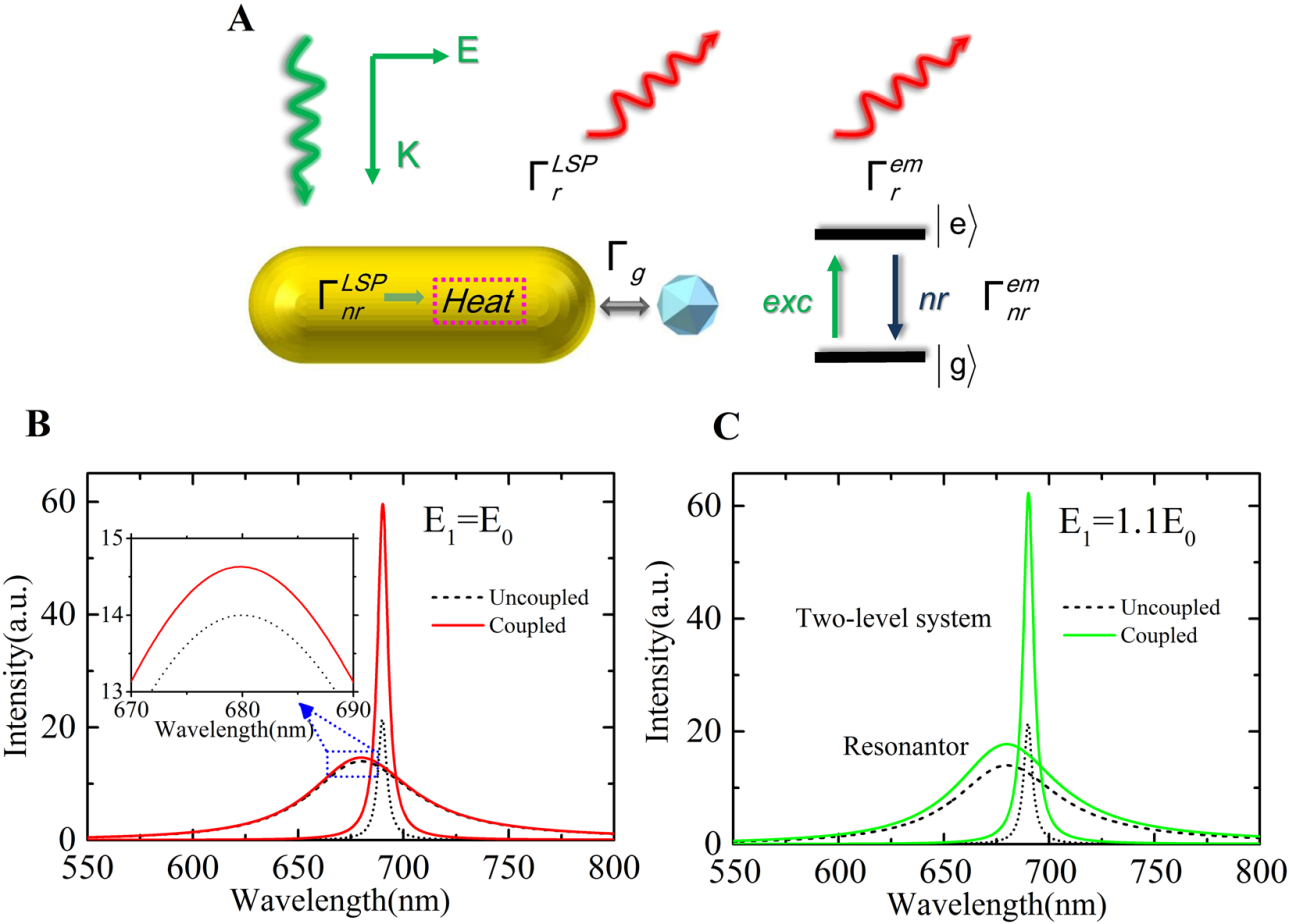
Scheme of the interaction between a FND and a GNR and theoretical analysis of the influence of the dipole coupling effect and local field of the dielectric particle. Simulated spectra of a resonator (black dash) and an atom (black dot) for *g* = 0, and corresponding spectra (solid curves) after coupling for *g* = 10 *meV* (**A**) the applied electromagnetic field *E* induces polarisations that causes dipole–dipole coupling Γ_*g*_ between the resonator and the atom and light emission ${\Gamma }_{r}^{LSP}$ from the GNR including both elastic and inelastic radiation processes. (**B**) Local field felt by the GNR for *E*_1_ = *E*_0_, i.e., without the dielectric nanoantenna effect (**C**) for *E*_1_ = 1.1*E*_0_ owing to the induced field of the polarised FND. The inset in (**B**) presents an enlarged view of the enhanced emission of the resonator.

To understand the physical origin of the enhancement of direct emission from the gold nanoparticles, we give a theoretical description of the interactions based on the concept of a quantised optical cavity, as shown in Fig. 3A. The GNR and FND hybrid was modelled as an entity consisting of a two-level atom and a nanoresonator cavity. We considered a FND interacting with a GNR separated by a distance. There is no direct electron tunnelling between the GNR and FND. The coupling mechanism is owing to dipole–dipole interaction. The artificial hybrid system is excited with a quasi-continuous-wave laser beam with frequency *ω*_*ex*_ polarised along the system axis. Considering the GNR as a plasmonic resonator, the Hamiltonian of the LSP resonator with mode *a* at *ω*_*c*_ is described as *H*_*c*_ = *ω*_*c*_*a*^†^*a*. Considering the FND as a two-energy-level atom system, the Hamiltonian is written as $${H}_{m}={\omega }_{g}\,|g\rangle \langle g|+{\omega }_{e}|e\rangle \langle e|$$, in which the energy difference between $$|g\rangle $$ and $$|e\rangle $$ is *ω*_*em*_ = *ω*_*e*_−*ω*_*g*_. Specifically, we define *σ*_−_ = $$|g\rangle $$$$\langle e|$$,*σ*_+_  = $$|e\rangle $$$$\langle g|$$ as the transition operators. Therefore, the free Hamiltonian of the whole system without any interaction is written as: *H*_0_ = *H*_*c*_ + *H*_*m*_ = *ω*_*c*_*a*^†^*a* + *ω*_*g*_$$|g\rangle $$ $$\langle g|$$ + *ω*_*e*_$$|e\rangle $$ $$\langle e|$$. The interaction Hamiltonian is described as: $${H}_{I}=g({a}^{\dagger }{\sigma }_{-}+a{\sigma }_{+})+{\mu }_{1}{E}_{1}({a}^{\dagger }{e}^{-i{\omega }_{ex}t}+a{e}^{i{\omega }_{ex}t})+{\mu }_{2}{E}_{2}({\sigma }_{+}{e}^{-i{\omega }_{ex}t}+{\sigma }_{-}{e}^{i{\omega }_{ex}t})$$, in which the first term demonstrates that the LSP mode couples with states $$|g\rangle $$ and $$|e\rangle $$, and *g* is the coupling constant. The second and the third terms show that the excitation electromagnetic field couples with the LSP mode and states $$|g\rangle $$ and $$|e\rangle $$, respectively. *μ*_1_ and *μ*_2_ are the corresponding coupling constants. *E*_1_ and *E*_2_ are the respective localised electromagnetic field amplitudes that the GNR and the atom feel. Hence, the Hamiltonian of this system is given by *H* = *H*_0_ + *H*_*I*_. The dynamics of these modes can be solved by the equations $$\dot{a}=i[H,a]-\kappa a=(-i{\omega }_{c}-\kappa )a-ig{\sigma }_{-}-i{\mu }_{1}{E}_{1}{e}^{-i{\omega }_{ex}t}$$ and $${\dot{\sigma }}_{-}=i[H,{\sigma }_{-}]$$ $$-\,\gamma {\sigma }_{-}=(-i{\omega }_{em}-\gamma ){\sigma }_{-}+iga{\sigma }_{z}+i{\mu }_{2}{E}_{2}{\sigma }_{z}{e}^{-i{\omega }_{ex}t}$$, where *κ* and *γ* are the total decay of the cavity and the atom, respectively. *σ*_*z*_ = $$|e\rangle $$ $$\langle e|$$ − $$|g\rangle $$ $$\langle g|$$, which represents the difference between the level occupation numbers of states $$|e\rangle $$ and $$|g\rangle $$. Details of the solution are shown in the supplemental materials. We assume that $$g\ll k-\gamma $$ because the SEF process is a weak coupling system^37,40–42^. This is supported by the experimental data, whereby the frequency of the zero-phonon line of the FND does not shift after coupling with the GNR.

For *g* = 0, which suggests no coupling between the GNR and FND, we obtained two Lorentz-shape spectra, a narrow peak for the atom and a broad band for the nanoresonator. When the coupling effect occurs (e.g., *g* = 10 *meV*) and the atom undergoes an enhanced excitation field owing to the plasmonic near field of the nanorod, then it is set as *E*_2_ = 1.5*E*_0_. At first, we assume that the presence of the nanodiamond does not influence the local field felt by the GNR, i.e., *E*_1_ = *E*_0_. Then, the emission from the nanoresonator increases slightly (less than 10%), as shown in Fig. 3B. This can be explained that total dipole moment of the NV cements in the FND is smaller than that of the GNP which resulting in low energy transfer rate, and that the inelastic plasmon radiation efficiency of the GNP is also very low. Hence the plasmon emission intensity due to the energy coupling from the FND should be low leading to small increasing. However, the enhancement of direct emission from the GNP can be over 40% in the above experiment. Then, the coupling effect cannot fully explain the total change. Therefore, we increased *E*_1_ and set it as*E*_1_ = 1.1*E*_0_, i.e., the presence of the FND increases the local field felt by the GNR. We obtained a considerable enhancement of the nanoresonator emission, as shown in Fig. 3C. This implied that the induced field produced by the polarisation of the FND increased the local field intensity felt by the GNR. A dielectric nanoparticle (DNP) as a non-plasmonic nanoantenna should be the main factor for the enhanced emission from a gold nanoparticle^43^. More details about the theoretical model is discussed in the supplemental materials.

**Figure 4:**
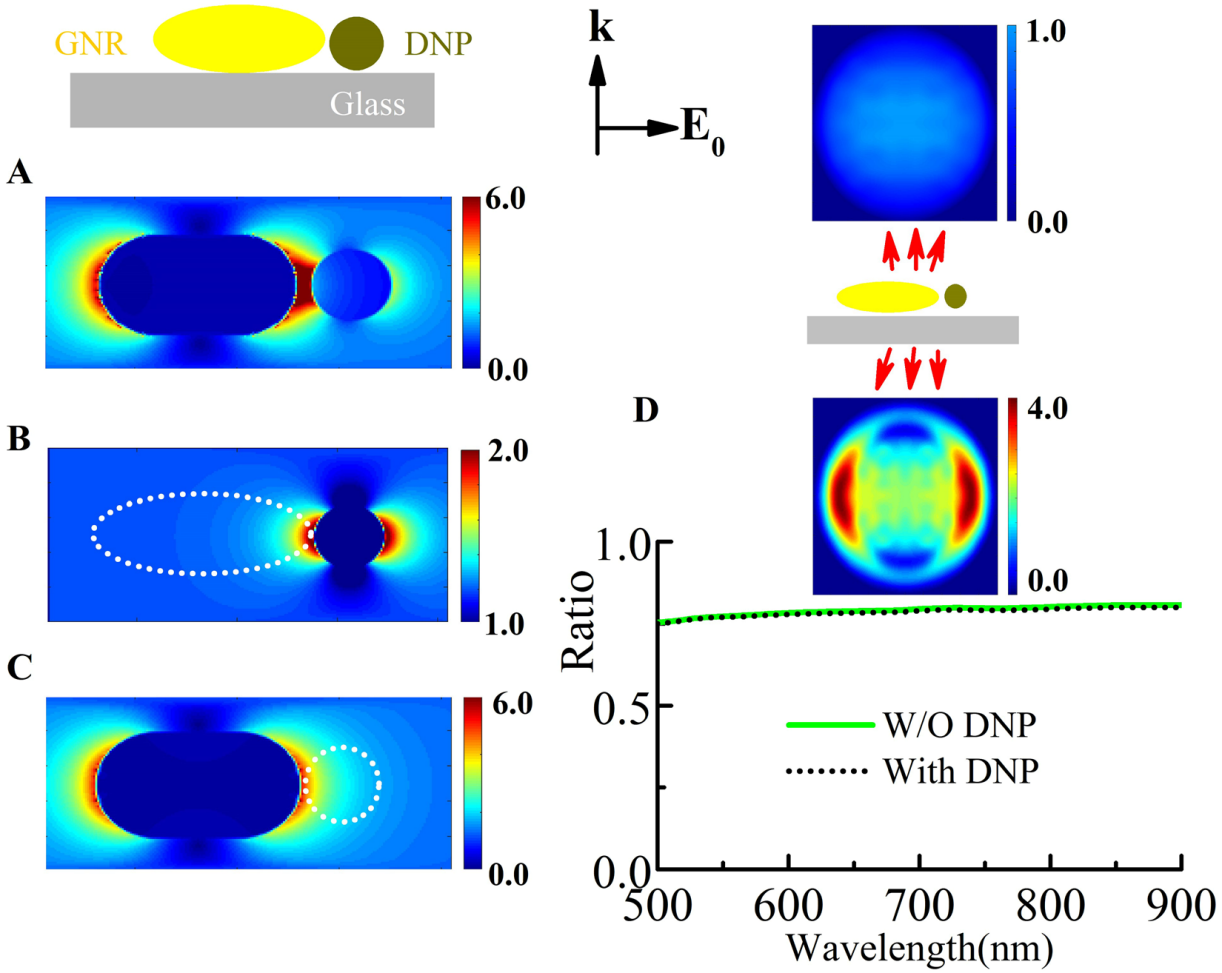
Field distributions (X–Z plane) of (**A**) a GNR with a DNP, (**B**) only a DNP, and (**C**) only a GNR. (**B**) The DNP with a diameter of 40 nm and refractive index of 2.3 in the FDTD calculations; the averaged field felt by the GNR (white dot range) is higher than the external excitation filed. (**C**) Near-field EM distribution around a GNR (60 nm×120 nm) at a wavelength of 532 nm. The white dot range shows the region of the DNP. (**D**) Ratio of emission flux from a GNR toward a glass substrate to total flux with (black dot) or without (green solid) the DNP as a function of wavelength. The inset in (**D**) shows representative emission patterns toward a glass substrate or air as indicated at a wavelength of 680 nm.

The dielectric nanoantenna effect is supported by the finite-difference time-domain (FDTD) numerical calculations shown in Fig. 4A–C. The DNP presents an enhanced local field that can increase the EM field felt by the GNR. Hence, in the SEF process, the light emission from the atom and nanoresonator both increase simultaneously. The plasmonic antenna effect and the dielectric nanoantenna effect contribute to this mutual enhancement. The influence of radiative directivity on the light collection efficient was negligible. As shown in Fig. 4D, the emission patterns of a GNR was simulated using the FDTD method. The patterns were almost the same before and after coupling with a DNP. The presence of DNP (i.e., increasing of the effective index for upper half-space) decreases the electromagnetic flux toward the glass substrate by less than 1% compared with that of the free GNR. So far, the theoretical results reveal that the plasmonic coupling effect can significantly enhance light emission from the emitter; it also results in a slight enhanced emission from the GNPs through the plasmon decay radiation. The enhancement of local field felt by the GNP should dominate the enhanced emission from the gold nanoparticles.

## Conclusion

In summary, we have demonstrated experimentally and theoretically that gold nanoparticles and fluorescent nanodiamonds mutually enhance their light emission when they are coupled for the SEF process. The light emission of the metal nanostructures should be considered for quantifying the SEF enhancement factor and for understanding the SEF spectral shape. These findings contribute to full and deep understanding of the SEF process. The SEF background arising from the metallic nanostructure is not noise or a constant background. The light emission from the metallic nanostructure is another indicator of the interaction strength between the metallic nanostructure and the emitter. The observation introduces a new perspective in the field of surface-enhanced spectroscopy to fully understand SEF process. For instance, the mutual interaction is also effective in surface-enhanced Raman scattering^44^. The coupling system for surface-enhanced spectroscopy should be considered as a hybrid entity to analyse and optimise.

## Materials and Methods

In our experiments, FNDs with a nominal size of 35 nm were prepared by radiation damage of type-Ib diamond powders containing several nitrogen vacancy centers^45^. The GNPs with diameter of approximately 80 nm and GNRs with size of approximately 60 nm × 100 nm were synthesised through a seed-mediated wet chemical method^46^. A dilute aqueous solution containing both the FNDs and GNRs was cast onto a silane-functionalised glass coverslip. Then, the nanoparticles were immobilised, with an average spacing of several micrometres for single particle-level investigations. We characterised the system optically with a micro-spectroscopy system using an inverted optical microscope combined with an AFM, as shown schematically in Fig. 1A. A continuous-wave laser at a wavelength of 532 nm passing through an objective lens was used to excite the samples, and the fluorescent emission was recorded through the same objective lens. The light emission was recorded *in situ* before and after coupling. Moreover, the fluorescence signal from the same particles could be switched to an avalanche photodiode, the lifetime and time trace were analysed with a TCSPC module (PicoHarp 300, PicoQuant). In this case, a picosecond laser diode operating at a wavelength of 480 nm with a repeat rate of 10 MHz was implemented for the fluorescence lifetime measurements. By AFM nanomanipulation, the GNPs were moved to approach the FNDs step-by-step, as shown in Fig. 1B^31,47–50^. In addition, the scattering spectra of the same GNPs was obtained *in situ* by the white light total internal reflection dark field method. Furthermore, the three-dimensional FDTD method was employed to simulate the emission flux, emission patterns, and electromagnetic field distribution of the nanostructures^51^. The calculations of the far-field radiation pattern were based on the near-field-to-far-field (NFTFF) transformation method. For the NFTFF calculations, we chose a large transformation plane (2 μm × 2 μm) placed 20 nm beneath the air/glass interface to collect most of the flux directed to the substrate. In the simulations, the mesh size was 1 nm to match the memory resources and computation time. The optical dielectric function of gold was modelled using the Drude–Lorentz dispersion model^52^. The refractive indices of the material were set to 1.0 for air and 1.49 for the glass substrate.

## Electronic supplementary material

Supplementary materials
