# Supplementary material for: Light Emission from Plasmonic Nanostructures Enhanced with Fluorescent Nanodiamonds
Source: Sci Rep. 2018 Feb 26;8:3605. doi: 10.1038/s41598-018-22019-z (PMC5826936; doi:10.1038/s41598-018-22019-z)
Supplement: Supplementary file 1 — Supplementary materials [file 41598_2018_22019_MOESM1_ESM.pdf]

Supplementary Information

Light Emission from Plasmonic Nanostructures Enhanced with Fluorescent Nanodiamonds

Jingyi Zhao,[1,†] Yuqing Cheng,[1,†] Hongming Shen,[1] Yuen Yung Hui,[2] Te Wen,[1] Huan-Cheng Chang,[2] Qihuang Gong,[1,3] and Guowei Lu[1,3,]*

*Corresponding author. E-mail: guowei.lu@pku.edu.cn

[1] State Key Laboratory for Mesoscopic Physics & Collaborative Innovation Center of Quantum Matter, Department of Physics, Peking University, Beijing 100871, China
[2] Institute of Atomic and Molecular Sciences, Academia Sinica, Taipei, 104 Taiwan, China
[3] Collaborative Innovation Center of Extreme Optics, Shanxi University, Taiyuan, Shanxi 030006, China

1. Coupling of a gold nanorod with a fluorescent nanodiamond

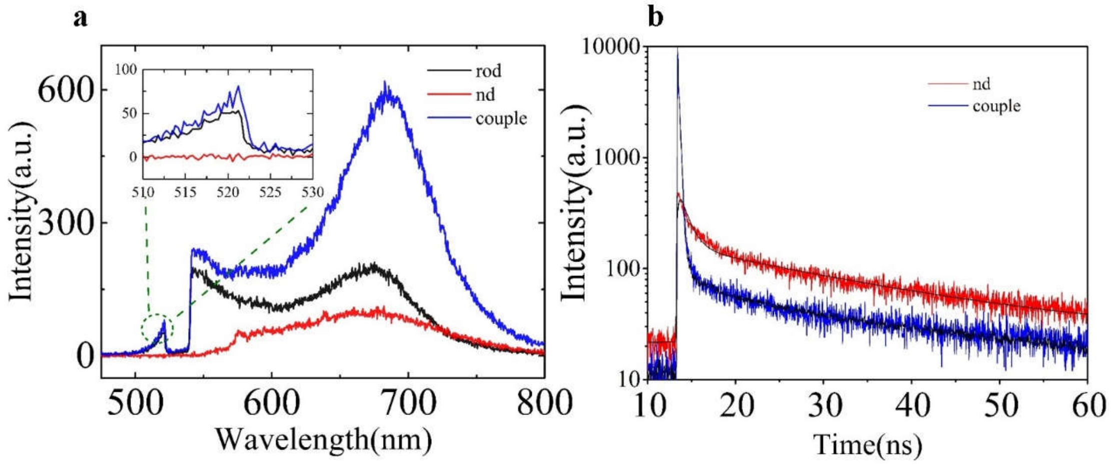

Figure S1. Experimental results of the coupling of a gold nanorod (GNR) with a fluorescent nanodiamond (FND). (**a**) Photoluminescence (PL) spectra of a free GNR (black) and a free FND (red) before coupling and the GNR–FND hybrid (blue) after coupling. The inset shows a magnification of those are showing the anti-Stokes component. (**b**) Fluorescent lifetime curves of the FND before coupling (red) and the FND-GNR hybrid after coupling (blue).

2. Polarization-dependent SEF process of the nanodiamond and nanorod hybrid system

In our experiments, both the gold nanorods and fluorescent nanodiamonds present excitation polarization-dependent characteristics. As the AFM tip moved the GNR to approach the FND, the orientation of the GNR was different after coupling in comparison to that before coupling. Moreover, when the assembly process was completed, it is reasonable to find proper polarization of the incident laser to achieve the maximum light emission from the hybrid system. Therefore, before the nano-manipulation process began, we recorded the spectra of the free GNR and FND under different excitation polarizations, as shown in Figure S2. For the discussion of the anti-Stokes emission enhancement, the overall spectra of the GNR before and after coupling with a nanodiamond under various polarizations were recorded. The maximum spectra of two series spectra were selected out for comparison, which can exclude the possibility that the enhancement of the anti-Stokes range is caused by alteration of the GNR's orientation.

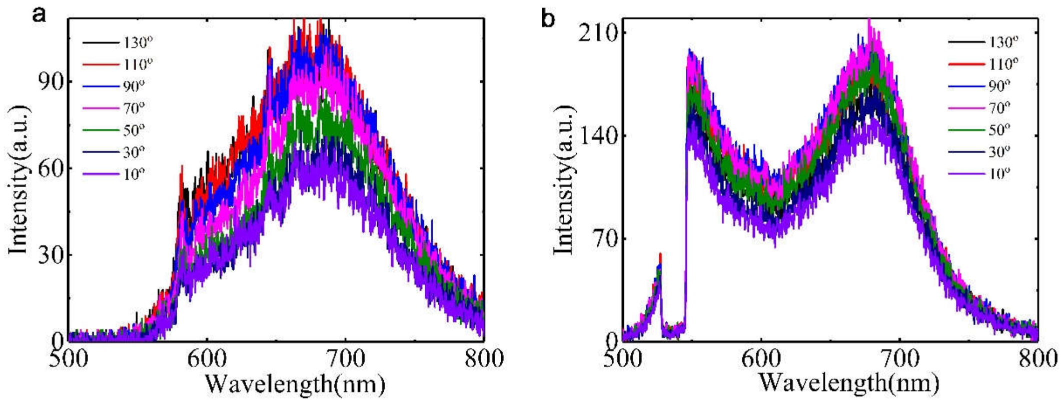

Figure S2. Experimental PL spectra of a free FND (**a**) and a free GNR (**b**) under various polarizations.

Moreover, during the long-time continuous measurements, the state of the instruments may change because of fluctuations in temperature or laser power, a shift in light focus position, or other unknown factors. We chose a reference point to calibrate the variation of the instrument conditions before and after coupling. As shown in Figure S3, rf0 was recorded before the approaching process and rf1 represents the state afterwards. When calculating the enhancement factor, this calibration coefficient was also taken into account.

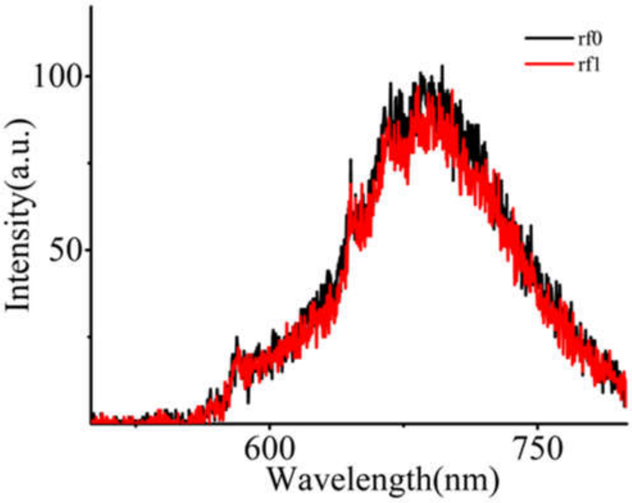

Figure S3. PL spectra of a reference point before (black) and after (red) coupling

3. SEF results of nanodiamond coupling with gold nanoparticle

The gold nanoparticle shows a LSP peak closer to the excitation wavelength (532 nm) than that of the gold nanorods. In this study, gold nanoparticles were synthesized by the citrate reduction method.[1] We obtained the relative spectra of the three components by fitting the SEF spectrum using $S_{SEF} = I_1 S_{GNR0} + I_2 S_{FND0} + g_c \hat{S}_{SCA} \hat{S}_{FND0}$; the result is shown in Figure S4, in which the PL direct emission from the GNP and the FND increase by 30% and 5%, respectively, and the indirect emission caused by the antenna effect makes up approximately 13% of the whole SEF spectra. Moreover, we compared the lifetime of the FND before and after coupling, as shown in the inset, and found that the FND lifetime was also shortened after coupling with the gold sphere.

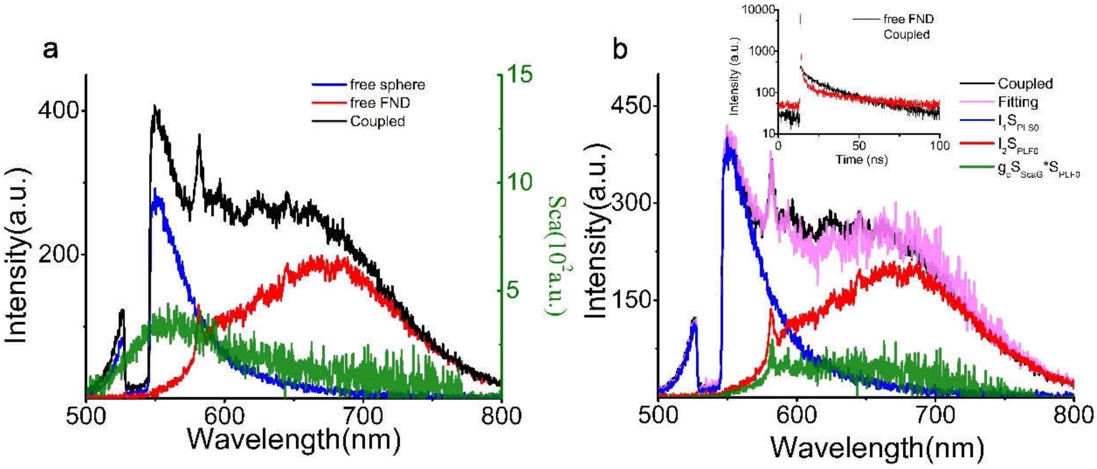

Figure S4. Analysis of the SEF results of nanoparticle coupling with FND (**a**) PL spectra for a sphere (blue) and a FND (red) before coupling and the SEF spectrum (black) after coupling. The scattering spectrum (green) after coupling is also shown. (**b**) Fitting spectra of $I_1 S_{GNR0}$, $I_2 S_{FND0}$, and $g_c \hat{S}_{SCA} \hat{S}_{FND0}$. The inset shows the lifetime of the FND before (black) and after (red) coupling with the sphere.

4. Theoretical model

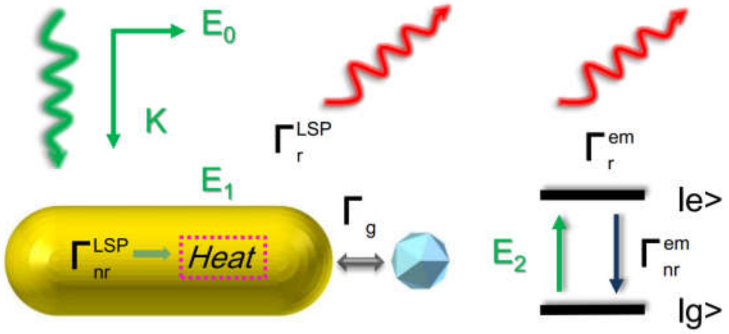

Figure S5. Scheme of the interaction between a FND and a GNR: the applied electromagnetic field E_0 induces polarizations that causes dipole–dipole coupling Γ_g, light emission Γ_r^{LSP} from the GNR including both elastic and inelastic radiation processes; Γ_{nr}^{LSP} represents the nonradiative decay rate. The effective local fields felt by the GNR and FND are E_1 and E_2, respectively.

Here, we employ a model considering a two-energy-level atom system as the emitter coupled with a plasmonic nanostructure which can be considered an optical nanocavity. [2] Fig. S5 shows a schematic diagram of the model. The GNR is considered a cavity with a resonance frequency ω_c and a total decay rate κ. The FND is considered a two-energy-level atom with energy levels |g> and |e>. A quasi-continuous-wave laser beam with frequency ω_{ex} couples with the ground state |g> and the excited level state |e>, whereby the energy difference between |g> and |e> is $\omega_{em} = \omega_e - \omega_g$. Regarding the GNR plasmonic resonator, the free energy of the LSP mode a with resonance frequency ω_c is described as $H_c = \omega_c a^\dagger a$. For the free atom, the Hamiltonian is written as $H_m = \omega_g |g><g| + \omega_e |e><e|$. Specifically, we define $\sigma_- = |g><e|$, and $\sigma_+ = |e><g|$ as the transition operators. Therefore, the free Hamiltonian of the system without any interaction is written as

$$H_0 = H_c + H_m = \omega_c a^\dagger a + \omega_g |g><g| + \omega_e |e><e| \quad (1)$$

In addition, the interaction Hamiltonian is described as

$$H_I = g(a^\dagger \sigma_- + a\sigma_+) + \mu_1 E_1 \left(a^\dagger e^{-i\omega_{ex}t} + a e^{i\omega_{ex}t}\right) + \mu_2 E_2 (\sigma_+ e^{-i\omega_{ex}t} + \sigma_- e^{i\omega_{ex}t}) \quad (2)$$

in which the first term suggests that the LSP mode is coupled with states |g> and |e>, and g is the coupling constant. The second and third terms describe the process in which the excitation electromagnetic field is coupled with the LSP mode and states |g> and |e>, respectively, and μ_1 and μ_2 are the respective coupling constants. E_1 and E_2 are the respective localised electromagnetic field amplitudes that the GNR and the atom feel. Hence, the Hamiltonian of this system is given by

$$H = H_0 + H_I \quad (3)$$

The dynamics of these modes can be solved by the equation

$$\dot{a} = i[H, a] - \kappa a = (-i\omega_c - \kappa)a - ig\sigma_- - i\mu_1 E_1 e^{-i\omega_{ex}t} \quad (4)$$

$$\dot{\sigma}_- = i[H, \sigma_-] - \ \gamma\sigma_- = (-i\omega_{em} - \gamma)\sigma_- + iga\sigma_z + i\mu_2 E_2 \sigma_z e^{-i\omega_{ex}t} \quad (5)$$

where κ and γ are the total decay of the GNR and the atom, respectively. $\sigma_z = |e><e| - |g><g|$, which represents the difference between the level occupation numbers of state |e> and |g>.

These equations can be solved rigorously, and the formal solutions are:

$$a = A_1 e^{(-i\omega_1 - \kappa_1)t} + A_2 e^{(-i\omega_2 - \kappa_2)t} + A_3 e^{-i\omega_{ex}t} \quad (6)$$

$$\sigma_- = B_1 e^{(-i\omega_1 - \kappa_1)t} + B_2 e^{(-i\omega_2 - \kappa_2)t} + B_3 e^{-i\omega_{ex}t} \quad (7)$$

where $\omega_1,\ \omega_2$ and $\kappa_1,\ \kappa_2$ are given as below:

$$\omega_1,\ \omega_2 = -\frac{1}{2} Im(D_1 \mp D_2), \text{ and } \kappa_1,\ \kappa_2 = -\frac{1}{2} Re(D_1 \mp D_2),$$

where we define $D_1 = -\kappa - \gamma - i\omega_c - i\omega_{em}$ and $D_2 = \sqrt{[(\kappa - \gamma) + i(\omega_c - \omega_{em})]^2 + 4\sigma_z g^2}$.

The coefficients A_1 and A_2 are complex amplitudes of modes $\omega_1,\ \omega_2$ for particle operator a. B_1 and B_2 are the complex amplitudes of modes $\omega_1,\ \omega_2$ for molecule operator σ_-. A_3 and B_3 are the complex amplitudes of scattering modes for a and σ_-, respectively.

For a weak coupling system, we assume that $g \ll \kappa - \gamma$; thus, the eigenfrequencies turn into $\omega_1,\ \omega_2 \approx \omega_c, \omega_{em}$, and the decay rates turn into $\kappa_1,\ \kappa_2 \approx \kappa, \gamma$. In the case of weak coupling, we ignore all the g^2 items and obtain these coefficients:

$$A_1 = \frac{iE_1\mu_1}{i(\omega_c - \omega_{ex}) + \kappa} - \frac{gE_2\mu_2\sigma_z}{[i(\omega_c - \omega_{ex}) + \kappa][i(\omega_c - \omega_{em}) + \kappa - \gamma]}$$

$$A_2 = \frac{gE_2\mu_2\sigma_z}{[i(\omega_{em} - \omega_{ex}) + \gamma][[i(\omega_c - \omega_{em}) + \kappa - \gamma]]}$$

$$B_1 = \frac{gE_1\mu_1\sigma_z}{[i(\omega_c - \omega_{ex}) + \kappa][i(\omega_c - \omega_{em}) + \kappa - \gamma]}$$

$$B_2 = \frac{iE_2\mu_2\sigma_z}{i(\omega_{em} - \omega_{ex}) + \gamma} - \frac{gE_1\mu_1\sigma_z}{[i(\omega_{em} - \omega_{ex}) + \gamma][[i(\omega_c - \omega_{em}) + \kappa - \gamma]]}$$

Using the input–output relation $\langle a_{out} \rangle = \sqrt{2\kappa_{ex1}} \langle a \rangle$, where $\langle \hat{Q} \rangle$ is the quantum average of the operator $\hat{Q}$, and κ_{ex1} is the outgoing coupling rate of the GNR, the detected intensity of light emission from the nanoparticle can be evaluated as

$$I_{full-a}(\omega) = \Re\left[\int_0^\infty \langle a_{out}^\dagger(\tau + t)a_{out}(t)\rangle e^{-i\omega\tau}d\tau\right] = \Re\left[\int_0^\infty \left[\frac{1}{T}\int_0^T a_{out}^\dagger(\tau + t)a_{out}(t)dt\right] e^{-i\omega\tau}d\tau\right] \quad (8)$$

in which $\Re[Q]$ stands for the real part of Q. $I_{full-a}(\omega)$ includes PL $I_{PL-a}(\omega)$ and scattering $I_{SC-a}(\omega)$. After filtering the input laser field and using the quantum regression theorem, we obtain the PL intensity of the nanoparticle from $I_{full-a}(\omega)$ as

$$I_{PL-a}(\omega) = 2\kappa_{ex1}\left(\frac{1-e^{-2\kappa T}}{2\kappa T}\right)\left[|A_1|^2\frac{\kappa}{(\omega-\omega_c)^2+\kappa^2} + |A_2|^2\frac{\gamma}{(\omega-\omega_{em})^2+\gamma^2}\right] \quad (9)$$

Similarly, using the relation $\langle\sigma_{out}\rangle = \sqrt{2\kappa_{ex2}}\langle\sigma_-\rangle$, in which κ_{ex2} is the outgoing coupling rate of the atom and T is the effective interaction time of the quasicontinuous-wave laser wave packet with the electrons, the detected intensity of the light emission from the atom can be evaluated as

$$I_{full-\sigma}(\omega) = \Re\left[\int_0^\infty \langle\sigma_{out}^\dagger(\tau + t)\sigma_{out}(t)\rangle e^{-i\omega\tau}d\tau\right] = \Re\left[\int_0^\infty \left[\frac{1}{T}\int_0^T \sigma_{out}^\dagger(\tau + t)\sigma_{out}(t)dt\right] e^{-i\omega\tau}d\tau\right] \quad (10)$$

and the PL intensity of the atom is obtained from $I_{full-\sigma}(\omega)$ as

$$I_{PL-\sigma}(\omega) = 2\kappa_{ex2}\left(\frac{1-e^{-2\gamma T}}{2\gamma T}\right)\left[|B_1|^2\frac{\kappa}{(\omega-\omega_c)^2+\kappa^2} + |B_2|^2\frac{\gamma}{(\omega-\omega_{em})^2+\gamma^2}\right] \quad (11)$$

Hence, the detected emission intensity spectrum would be

$$I_{PL}(\omega) = I_{PL-a}(\omega) + I_{PL-\sigma}(\omega) \quad (12)$$

So far, we have deduced the PL spectra from both the GNR and the atom with the coupling coefficient g. For an extreme case, $g = 0$, which suggests that there is no interaction between the GNR and the atom, and the coefficients would be given in the following form:

$$A_1 = \frac{iE_1\mu_1}{i(\omega_c-\omega_{ex})+\kappa},\ A_2 = 0,\ B_1 = 0,\ B_2 = \frac{iE_2\mu_2\sigma_z}{i(\omega_{em}-\omega_{ex})+\gamma}$$

The modes and decays are regressed to the original form:

$$\omega_1,\ \omega_2 = \omega_c, \omega_a,\ \ \kappa_1,\ \kappa_2 = \kappa, \gamma$$

Thus, the PL spectrum from each operator (a, σ_-) presents a single Lorentz line:

$$I_{PL-a}(\omega) = 2\kappa_{ex1}|A_1|^2\left(\frac{1-e^{-2\kappa_1 T}}{2\kappa_1 T}\right)\frac{\kappa_1}{(\omega-\omega_1)^2+\kappa_1^2} \quad (13)$$

$$I_{PL-\sigma}(\omega) = 2\kappa_{ex2}|B_2|^2\left(\frac{1-e^{-2\kappa_2 T}}{2\kappa_2 T}\right)\frac{\kappa_2}{(\omega-\omega_2)^2+\kappa_2^2} \quad (14)$$

which corresponds to the PL from a single free emitter (GNR or atom).

Employing the equations above, we can calculate the enhancement factor for both the GNR and atom. We define $M_1 = \frac{\kappa_{exa}}{\kappa_{exa}}\left|\frac{A_1(g\neq0)}{A_1(g=0)}\right|^2 + \frac{\kappa_{exb}}{\kappa_{exa}}\left|\frac{B_1(g\neq0)}{A_1(g=0)}\right|^2 = \left|\frac{E_1}{E_0} - \frac{gE_2\mu_2\sigma_z}{iE_0\mu_1[i(\omega_c-\omega_{em})+\kappa-\gamma]}\right|^2 + \frac{\kappa_{exb}}{\kappa_{exa}}\left|\frac{E_1}{E_0}\frac{g\sigma_z}{i(\omega_c-\omega_{em})+\kappa-\gamma}\right|^2$ to describe the enhancement factor for the GNR and $M_2 = \frac{\kappa_{exb}}{\kappa_{exb}}\left|\frac{B_2(g\neq0)}{B_2(g=0)}\right|^2 + \frac{\kappa_{exa}}{\kappa_{exb}}\left|\frac{A_2(g\neq0)}{B_2(g=0)}\right|^2 = \left|\frac{E_2}{E_0} - \frac{gE_1\mu_1}{iE_0\mu_2[i(\omega_c-\omega_{em})+\kappa-\gamma]}\right|^2 + \frac{\kappa_{exa}}{\kappa_{exb}}\left|\frac{E_2}{E_0}\frac{g}{i(\omega_c-\omega_{em})+\kappa-\gamma}\right|^2$ for the atom.

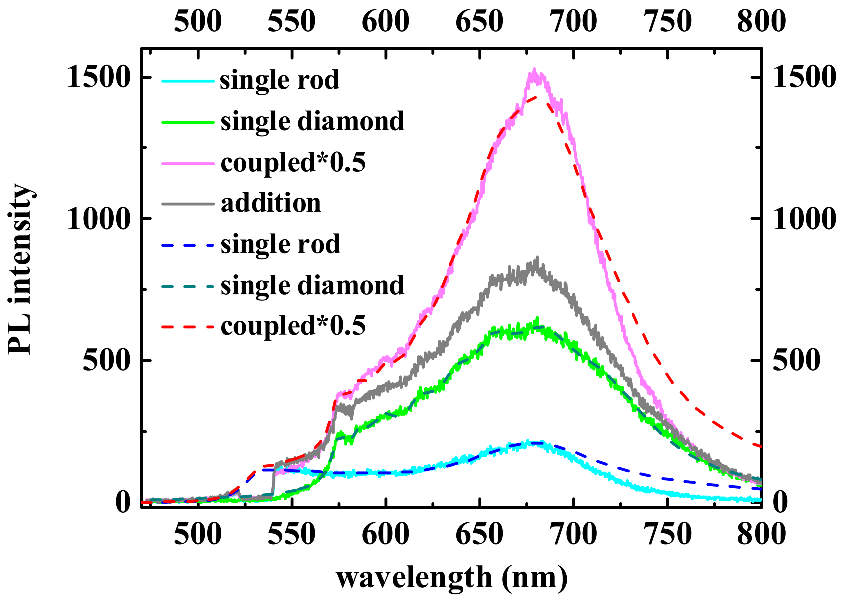

Figure S6. Experimental PL spectra (solid lines) and the fitting data (dashed lines). Blue, green, and pink solid lines show the experimental PL spectra of a single rod, single diamond, and their hybrid system, respectively. Blue, green, and red dashed lines show the fitted PL spectra of a single rod, single diamond, and coupled system, respectively. The grey line shows the sum of the blue and green solid lines.

To understand the experimental results, we fit one of the experimental SEF spectra by employing these calculations. Figure S6 shows the experimental lines and the fitting lines. The grey line shows the sum of the blue and green solid lines, for comparison. For clarity, the coupled spectrum is reduced by half because its intensity is much stronger than that of the single spectra. Here, the nanodiamond was assumed to be a system with multiple two-levels, without interaction between the two-levels. We consider it as an atom with 8 independent two-level systems, which will all couple with the GNR mode. Therefore, the nanodiamond is fitted with 8 Lorentz lines, with the corresponding frequency and decay rate. For the anti-Stokes part, the electron occupation in the *sp*-band near the Fermi level follows the Fermi–Dirac distribution, i.e., $\propto \frac{1}{1+e^{-\Delta E/k_B T}}$. The line-shape of the anti-Stokes emission is dominated by the thermal equilibrium electron distribution. Hence, the mutual enhancement mechanism is also effective for

such multiple-energy level systems. Here, to understand the anti-Stokes emission difference between the gold nanoparticles and fluorescent nanodiamond, we explain it as follow in simply: For gold nanoparticle, Fermi level is in the conduction band. Due to thermal effect, some electrons occupy higher energy levels over the Fermi level, these electrons could return immediately to near Fermi level after photon excitation, resulting in anti-Stokes emission.[3, 4] Regarding to luminescent centers nanodiamond, although there should be also thermal distribution for the electrons at ground states, because there is strong electron phonon coupling effect, the photon excited electrons would decay to lowest excited vibronic levels irrespective initial excited energy, then transition to ground states. Hence, it is unlikely to appear anti-Stokes emission for the fluorescent nanodiamond in present study.

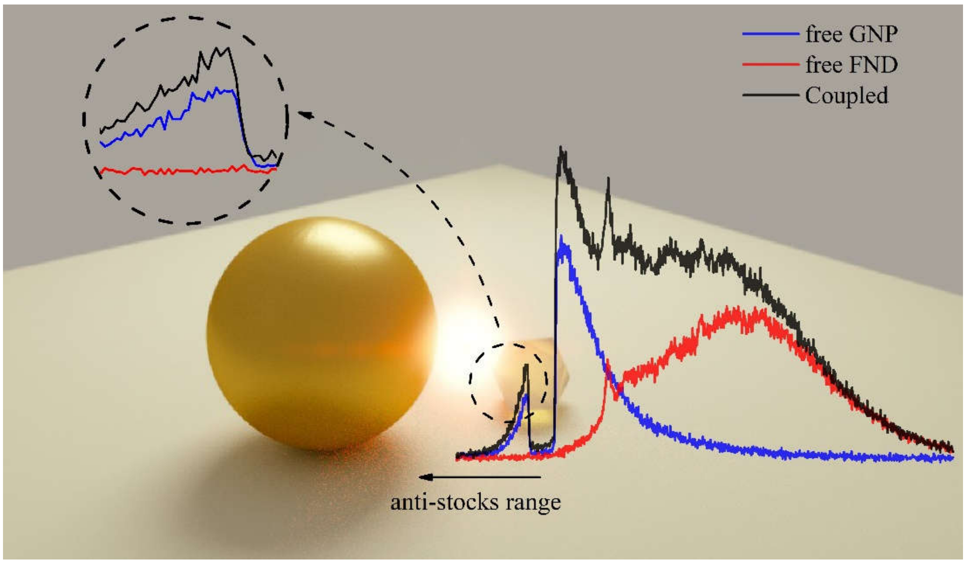

Figure S7. Table of contents. The plasmonic nanoparticles and fluorescent nanodiamonds mutually enhance their light emission when they were coupled together for the surface enhanced fluorescence process. The enhancement of local field due to dielectric antenna effect dominates the light emission enhancement of the plasmonic nanoparticles.

Reference

1. Q. Wang, G. Lu, L. Hou, T. Zhang, C. Luo, H. Yang, G. Barbillon, F. H. Lei, C. A. Marquette, P. Perriat, O. Tillement, S. Roux, Q. Ouyang and Q. Gong, *Chem Phys Lett*, 2011, **503**, 256-261.
2. K. Y. Xia, Y. B. He, H. M. Shen, Y. Q. Cheng, Q. H. Gong and G. W. Lu, *Proc SPIE, Micro+Nano Materials, Devices, and Systems*, 2015, **9668**, 96685B.
3. Y. He, K. Xia, G. Lu, H. Shen, Y. Cheng, Y. C. Liu, K. Shi, Y. F. Xiao and Q. Gong, *Nanoscale*, 2015, **7**, 577-582.
4. A. Carattino, V. I. Keizer, M. J. Schaaf and M. Orrit, *Biophys J*, 2016, **111**, 2492-2499.
